# Application of growth models to South African Boer goat castrates and does under feedlot conditions

**DOI:** 10.1007/s11250-024-03973-5

**Published:** 2024-05-29

**Authors:** T. S. Brand, J. P. van der Westhuyzen, W. Hough, J. H. C. van Zyl

**Affiliations:** 1 Department of Animal Sciences, Stellenbosch University, Private Bag X1, Matieland, Stellenbosch, 7602 South Africa; 2 Directorate: Animal Sciences, Department of Agriculture, Western Cape Government, Private Bag X1, Elsenburg, 7607 South Africa

**Keywords:** Brody, Gompertz, Inflection point, Logistic, Von Bertalanffy

## Abstract

Mathematical models may aid researchers in describing biological processes, like growth, in animals. This study aimed to collect the body weight data of 18 Boer goat castrates and 20 Boer goat does, from birth until maturity, to model growth and determine growth trends. This is a novel investigation as sufficient information on an age-weight database for these two Boer goat sexes from birth to maturity, is lacking. Using age-weight data, four nonlinear models, namely the Brody, Gompertz, Logistic and Von Bertalanffy growth models, were plotted and evaluated. The model parameters of each growth model were compared for differences between the two sexes. The statistical effectiveness of fit was determined for each model using AIC and RMSE, with R^2^ also being considered. All models except the Brody model, predicted significantly heavier mature weights for castrates. The Brody model was deemed unfit to describe Boer goat growth as the function severely over-predict weights from birth until maturity for both sexes. The Von Bertalanffy (R^2^ = 91.3) and Gompertz functions (R^2^ = 91.3) showed the best fit for Boer goat castrates, while the Gompertz model (R^2^ = 95.1) showed the best fit for Boer goat does. The Gompertz function is the preferred model to depict Boer goat growth overall, as it accurately characterized growth of both sexes. According to the Gompertz model the age at which the inflection point of the growth curve was reached, did not differ significantly between castrates and does (141.80 days versus 136.31 days). There was also no significant difference in maturation rate between the two sexes.

## Introduction

Growth is a key process in animal development and is perhaps the most specific and varied attribute investigated, encompassing many, if not all, levels of the individual phenome (Stass 2017). Growth models typically condense age-weight data, from birth until maturity, into parameters that can be interpreted biologically, to aid in deciphering animal growth patterns (Bathaei and Leroy 1996; Norris et al. 2007; da Silva et al. 2012). Animal growth normally follows a sigmoidal curve (Tariq et al. 2013), therefor nonlinear models are usually used to plot growth of mammals (Curi et al. 1985; Franco et al. 2011; Silva et al. 2011). Growth initially occurs slowly, in a positive acceleration phase, then increases rapidly as the animal experiences a prepubescent self-accelerating rapid growth phase, approaching an exponential growth rate. Finally, an inflection point (maturity) is reached, from where the growth decreases gradually due to post-pubescent self-inhibition in what is known as a negative acceleration phase (Owens et al. 1993).

Several growth equations, including the Brody, Von Bertalanffy, Logistic, and Gompertz models, have been employed to plot livestock growth specifically. These models then have the capacity to represent weight gain and evaluate several relevant biological metrics, such as mature weight and maturation rate - valuable tools to predict the animal’s daily feed requirements and evaluate environmental effects on growth rate (Teleken et al. 2017). Numerous researchers have found that parameters estimated by the growth curve may be used in selection programs to influence the weight-age relationship beneficially (Fitzhugh 1976; Mukasa-mugerwa and Lahlou-Kassi 1995; Abegaz et al. 2010) and that growth trend parameters can successfully be employed in selection schemes as these traits are highly heritable (Merrit 1974; Menchaca et al. 1996; Şengül & Kiraz 2005). When growth models are analysed, optimal slaughter ages can also be determined (Malhado et al. 2009). As a result, the mathematical modelling of animal growth is regarded as a critical tool for the optimization of the animal production industry (Nadarajah et al. 1984; Schinckel & de Lange 1996).

The assessment of growth curves for different animal species is widespread throughout literature, describing the growth of pigs (Whittemore and Green 2001), cattle (López de Torre et al. 1992; Menchaca et al. 1996), sheep (Ghavi Hossein-Zadeh 2015; Tariq et al. 2013), poultry (Şengül and Kiraz 2005), and even unique farm animals such as the Bolivian llama (Wurzinger et al. 2005).

Continual increases in human population (FAOSTAT 2021) lead to an increased demand for animal protein, posing an opportunity for scarcely used red meat sources, like goats, to supply this demand. Goat farming is less common than sheep farming in South Africa, with an expected 11 510 tonnes of goat meat, also known as chevon, produced in 2019, while 160 750 tonnes of sheep meat was produced in the same year (FAOSTAT 2021). The goat population in South Africa decreased from 5 872 332 head of goats in 2015 to 5 251 039 in 2019, while goat meat production increased from 10 814 tonnes to 11 510 tonnes over the same period (FAOSTAT 2021). Boer goats exhibit a high degree of adaptability, which is a critical economic characteristic since it influences an animal’s production and profitability (Casey and van Niekerk 1988). Additionally, the breed is recognized for its superior growth rate over indigenous goats as well as a higher carcass yield (Asizua et al. 2014; Teklebrhan 2018; Visser 2019; Manirakiza et al. 2020). Boer goats therefore serve as the benchmark for other meat goat breeds (Steyn 2010). Breeders commonly utilise Boer goat bucks in crossbreeding schemes with native goats to increase growth rate and improve mature weight.

Since literature modelling the growth of goats, specifically the improved Boer goat, is lacking, models used to describe the growth of sheep are often used to estimate the growth of goats. García-Muñiz et al. (2019) found the Generalized Michaelis-Menten to best explain the growth of Boer goat does in the Mexican national breeding flock. Çak et al. (2017) proposed that the Gompertz and Richards functions best describes the Angora goat’s growth. The Logistic, Von Bertalanffy and Brody function has been described by de Freitas (2005) to model sheep growth accurately, while Lambe et al. (2006) suggested that the Richards and Gompertz model should also be considered. Other researchers have also proposed the use of the Brody model to plot the growth of Iranian fat-tailed sheep (Bathaei and Leroy 1996), Kivircik and Daglýc male lambs (Akbaş et al. 1999), and African dwarf sheep (Gbangboche et al. 2008). The Gompertz and Von Bertalanffy model suitably described growth in Morkaraman and Awassi lambs (Topal et al. 2004), while the Gompertz and Logistic models suitably fit the growth of local Brazilian and Dorper crosses (Malhado et al. 2009). Alizadeh (2015) and Eyduran et al. (2008), respectively found the Gompertz model most suitable for describing growth in Moghani sheep and Kivircik sheep.

With the increase in production of small stock, more producers are opting for intensive feedlot finishing systems that incorporate technology-based farm management systems (Banhazi et al. 2012), thus it has become essential to estimate the growth characteristics of unconventional livestock such as Boer goats. This will allow producers to predict the appropriate market and slaughter weight, given current market requirements and conditions.

It is common practice to finish sheep in feedlots as they reach market weight quicker due to an increased growth rate from high concentrate feeds and lack of energy expenditure (Leme et al. 2013). Since feedlots rely on growth, feed is a fundamental factor of profit. Feedlot nutrition thus focuses on supplying the animal with adequate amounts of energy and protein to ensure optimal growth with adequate feed intake and feed conversion (Wang et al. 2014). Sheridan et al. (2003) found that goats may better utilize roughage-based diets but are able to adapt to feedlot conditions where high concentrate-based diets are supplied. Goats tend to generally exhibit lower growth rates than sheep (Van Niekerk and Casey 1988) and deposit more abdominal fat rather than subcutaneous fat when compared to sheep (Casey and Webb 2010). These differences indicate a difference in maturation rate between the two species, and a need to characterise goat growth.

The objective of this study was to describe Boer goat growth in a feedlot system, using the Brody, Gompertz, Logistic and Von Bertalanffy nonlinear models. Furthermore, the study aimed to identify the model best suited to describe the growth patterns of Boer goat castrates and does.

## Materials and methods

The 38 Boer goat kids used in this study were obtained from the Nortier Research Farm near Lambert’s Bay (32°02’06.5’’S; 18°19’54.6’’E). The growth of 18 Boer goat castrates and 20 Boer goat does was observed for this study. Goat kids were identified within 24 hours of birth, with the sex and birth weight being recorded. To measure growth rate, the goat kids were weighed weekly from birth until maturity, at which point they were slaughtered. Male goat kids were castrated at one week of age. Weaning commenced when kids were 15 weeks old at an average weight of 25.6 ± 3.5 kg. After weaning, the Boer goat kids were transported to the Elsenburg Research Farm near Stellenbosch (33°50’42.9"S; 18°50’05.0’’E). Upon arrival at Elsenburg, all animals were vaccinated using a broad-spectrum vaccine for the prevention of *Pasteurella* and *Clostridia* and randomly allocated to individual pens for the trial. The kids were reared in individual pens, eliminating competition for feed resources, thus allowing maximal genetic growth potential. The kids received a ruminant feedlot diet (Tables 1 and 2) consisting of 10.52 MJ ME/kg energy and 14.81% protein on a dry matter basis. Goats were gradually adapted to the trial diet, by supplying roughage (oat hay) in addition to the trial diet and reducing roughage daily using a step-up program over 10 days. All animals had *ad libitum* access to the trial diet from weaning until slaughter.

**Table 1:** Composition of the trial diet provided to Boer goat kids

Ingredients	% As fed
Oat hay	37.74
Corn Yellow grain	38.83
Canola oilcake meal	14.56
Molasses powder	2.43
Bicarbonate of Soda	1.94
Salt	0.97
Limestone	0.97
Slaked Lime	0.48
Vitamin and Mineral premix	0.48
Urea	0.49
Ammonium Sulphate	0.49
Ammonium Chloride	0.49
Mono calcium phosphate	0.13

**Table 2:** Nutrient composition of the Boer goat trial diet on an as-fed and DM basis

Nutrients	As fed	Dry matter
Dry matter, %	90.03	100
Moisture, %	9.97	0
Total digestible nutrients (TDN), %	63.16	70.16
Metabolizable energy, MJ/kg feed	9.95	10.52
Protein, %	13.33	14.81
Fibre, %	13.98	15.53
Acid detergent fibre, %	16.57	18.40
Neutral detergent fibre, %	30.13	33.47
Calcium, %	0.96	1.07
Phosphorous, %	0.36	0.40

Statistica (version 14) was used to statistically analyse body weight data of the Boer goats at respective ages. The nonlinear estimation function was used to fit the growth functions to the growth curve of each goat from the respective sexes. All the growth models (Table 3) were plotted using the Gauss-Newton iteration method with step halving set to 100 iterations. Individual growth curves (18 castrate curves and 20 doe curves) were used to determine and establish parameter values for each corresponding growth model. Individual non-convergent curves were considered outliers and removed from the dataset.

**Table 3:** Growth model functions used to model the growth of Boer goat ewes and castrates

Model	Function	Reference
Brody	*W*_*t*_ *= A(1 - Be* ^*− kt*^ *)*	Brody 1945
Gompertz	${W}_{t}=A{e}^{{-e}^{-k(t-c)}}$	Emmans 1989
Logistic	*W*_*t*_ *= A/(1 + Be* ^*− kt*^ *)*	Nelder 1961
Von Bertalanffy	*W*_*t*_ *= A(1 – Be* ^*− kt*^ *)* ^*3*^	Von Bertalanffy 1957

*W*_*t*_ = goat’s body weight at time *t*; *A* = asymptotic mature weight of the goat; *B* = proportional live weight to be gained by the goat after birth; *k* = maturation rate; *C* in the Gompertz function = age of goat at inflection point

The respective growth model’s parameter values were then compared between the Boer goat sexes using the analysis of variance (ANOVA) procedure of Statistica. The influence of sex (castrate or doe) on the parameters of the respective growth functions was then evaluated. A 95% confidence level (*P* ≤ 0.05) was used to establish whether significant differences exist in model parameters due to the main effect of sex. All parameter values for the various growth models are stated as least square means (LSM) accompanied by the parameter’s standard error. The root means square error (RMSE) and Akaike information criterion (AIC) between the expected and observed values obtained from the growth models were used as goodness of fit statistics to determine which growth model most suitably described growth for each sex. The coefficient of determination (R^2^) was also calculated and used as an indication of the amount of variation explained by each model. After parameters were obtained, a correlation analysis was also performed on the parameters within each model to determine the relationship between the biological characteristics represented by the parameters.

## Results

For the construction of the growth models, each goat’s weekly weight-age data was plotted using the Brody, Gompertz, Logistic and Von Bertalanffy growth functions. The parameter means and standard errors are presented in Table 4. No difference due to the main effect of sex was found for any of the parameters for the Brody model, however, significant differences were found for the asymptotic mature weight (*A*) between the sexes for the Gompertz, Logistic and Von Bertalanffy growth functions (Table 4). The asymptotic mature weight (*A*) predicted by the models ranged from 75 to 194 kg for castrates and from 67 to 155 kg for does (Table 4). The Brody model predicted the heaviest asymptotic mature weight of all the growth models, estimating the mature weight of Boer goat castrates and does to be 194.17 kg and 155.46 kg, respectively. The mature weights predicted by the Brody model are also noticeably higher than the mature weights predicted by the other models. The asymptotic mature weights predicted by the Von Bertalanffy and Gompertz model closely resemble one another and only differ by ± 10 kg for the respective sexes. The Von Bertalanffy model predicted the second highest mature weights for Boer goat castrates and does at 102.7 kg and 88.8 kg, respectively. While the Gompertz model estimated the mature weights to be 90.2 kg and 79.3 kg for castrates and does, respectively. The Logistic model predicted the lowest mature weights at 75.8 kg for castrates and 69.8 kg for does. No significant differences were found between Boer goat castrates and does for the proportional weight to be gained after birth (parameter *B*) for any of the models (Table 4). The highest *B*-parameter value was obtained in the Logistic function (5.75 and 5.92 for castrates and does, respectively) and the smallest value by the Von Bertalanffy model (0.55 and 0.56 for castrates and does, respectively).

No difference (*P* > 0.05) in maturation rate (parameter *k*) due to the main effect of sex was found for any of the four growth models (Table 4). The models’ maturation rates (*k*) showed a negative correlation with the asymptotic mature weights (*A*). There seems to be a negative correlation between asymptotic mature weight and maturation rate. A negative correlation of *r*= -0.833 was obtained between parameter *A* and *k*, across all the models. The correlation between the asymptotic mature weight and maturation rate of castrates and does were *r* = -0.855 and *r* = -0.859, respectively. The Logistic model showed the fastest maturation rate (0.0104 and 0.0107 for Boer goat castrates and does, respectively) while the Brody model had the slowest maturation rate (0.0011 and 0.0013 for Boer goat castrates and does, respectively) (Table 4). Although the growth models show no significant difference between maturation rate between the sexes, castrates exhibited superior growth rates and higher mature weights.

The inflection point of a growth model is important since it depicts the age or weight at which the growth rate peaks. There seems to be a positive relationship between mature weight (parameter *A*) and the age at inflection (parameter *C*) (Table 4). To calculate the Logistic function’s inflection point the *A*-parameter of Boer goat castrates and does were halved and the model predicted that castrates would attain their inflection point at 37.91 ± 1.04 kg and does will attain their inflection point at 33.90 ± 0.99 kg (Table 4). This suggests that Boer goat castrates will attain maximal growth at 163–174 days of age while does will experience maximal growth between 160 and 171 days of age. With regards to the Gompertz model, *C* denotes the inflection point. No significant difference was found between the inflection point of Boer goat castrates and does. The Gompertz model estimated the curve’s inflection point to occur at approximately 141.80 days for Boer goat castrates and approximately 136.31 days for Boer goat does (Table 4). By using the formula from Emmans (1989) it is possible to deduce that Boer goat castrates and does reach maximal growth at a weight of 33.17 ± 1.22 kg and 29.17 ± 1.06 kg, respectively. In both growth models, Boer goat does attain their maximal growth earlier due to a slightly higher, however not significantly higher, maturation rate. The Logistic model predicted that both sexes would reach their inflection point at an older age as well as a heavier weight than the predictions from the Gompertz model.

**Table 4:** Comparison of the estimated growth parameters (mean ± S.E.) of the four growth models describing the growth of Boer goat castrates and does

Growth model		Sex		*P*-value
	Parameters*	Castrates	Does	
Brody	*A*	194.2 ± 30.56	155.5 ± 13.94	0.22
	*B*	0.962 ± 0.0043	0.961 ± 0.0025	0.88
	*k*	0.0011 ± 0.0002	0.0013 ± 0.0002	0.77
Gompertz	*A*	90.2^a^ ± 3.14	79.3^b^ ± 2.98	0.02
	*C*	141.80 ± 6.36	136.31 ± 6.04	0.54
	*k*	0.0058 ± 0.0002	0.0060 ± 0.0002	0.43
Logistic	*A*	75.8^a^ ± 2.08	67.8^b^ ± 1.97	0.01
	*B*	5.751 ± 0.192	5.923 ± 0.182	0.52
	*k*	0.0104 ± 0.0003	0.0107 ± 0.0003	0.38
Von Bertalanffy	*A*	102.7^a^ ± 4.49	88.8^b^ ± 4.25	0.03
	*B*	0.553 ± 0.007	0.556 ± 0.006	0.74
	*k*	0.0042 ± 0.0002	0.0044 ± 0.0002	0.47

*A = asymptotic mature weight of goat; *B* = proportional live weight to be gained by goat after birth; *k* = maturation rate; *C* In Gompertz function = age of goat at inflection point

Generally, the four growth functions fit the observed growth data well. Table 5 displays the evaluation of the statistical fit of each model, to establish which of the growth models most appropriately describes the growth of Boer goat castrates and does. Lower values for RMSE and AIC signify a better fit for the growth model to the data and a larger R^2^ percentage advocate for a more significant proportion of the variation in the data being explained by the growth model. For Boer goat castrates, the Logistic model was found to have the least effective statistical fit of the four growth models (Table 5), as the model had the highest RMSE and AIC as well as the lowest R^2^. This is expected as the Logistic model had noticeably lower asymptotic mature weights than the other models (Table 4). Furthermore, the Brody model displayed the lowest RMSE and AIC with the second best R^2^-value of 91.4%. The von Bertalanffy model had comparatively poor goodness of fit statistics for Boer goat castrates and a R^2^-value of 91.3%. The Gompertz model had slightly higher RMSE and AIC values than the von Bertalanffy model but had an equally good R^2^-value. Regarding Boer goat does, the Von Bertalanffy and Logistic model was the least accurate in modelling growth as the models had comparatively high RMSE and AIC values with noticeably lower R^2^-values compared to the Brody and Gompertz functions (Table 5). Presumably, this is because the Logistic model underestimated the asymptotic mature weight (parameter *A*) and the Von Bertalanffy model had slightly exaggerated asymptotic mature weights for does. The Gompertz model described the growth of Boer goat does the best by exhibiting the lowest RMSE and AIC values along with highest R^2^-value, with 95.1% of the variation in the data being explained by the model. The Brody model had an equally high R^2^-value but had slightly worse RMSE and AIC values. When comparing the asymptotic weight between the models (Table 4), it can be deduced that the Brody model will overestimate asymptotic mature weight (parameter *A*) for Boer goat castrates and does and should thus not be considered as a function to model Boer goat growth. Thus, although the Brody model showed a relatively good statistical fit, the biological significance is lacking. When considering the statistical goodness of fit, the Von Bertalanffy model most suitably describes the growth of Boer goat castrates. However, the Gompertz model also displayed a good fit and is sufficient in explaining the growth of Boer goat castrates as well as does and would thus be most suitable for describing the growth of these two sexes of Boer goats in general. The Logistic function showed the worst fit for the growth of Boer goat castrates and the Von Bertalanffy model fits Boer goat does’ growth the worst.

**Table 5:** Comparison of the statistical goodness of fit* for the Brody, Gompertz, Logistic and Von Bertalanffy models used to describe the growth of Boer goat castrates and does

Model	Castrate			Does		
	RMSE	AIC	R^2^	RMSE	AIC	R^2^
Brody	5.238	2836.931	91.4	3.645	2480.102	95.1
Gompertz	5.241	2838.000	91.3	3.633	2474.006	95.1
Logistic	5.266	2846.142	91.1	3.667	2491.616	94.9
Von Bertalanffy	5.237	2836.636	91.3	5.237	2836.636	91.3

*RMSE = root mean square error; AIC = Akaike’s information criterion; R^2^ = coefficient of determination

**Fig. 1:**
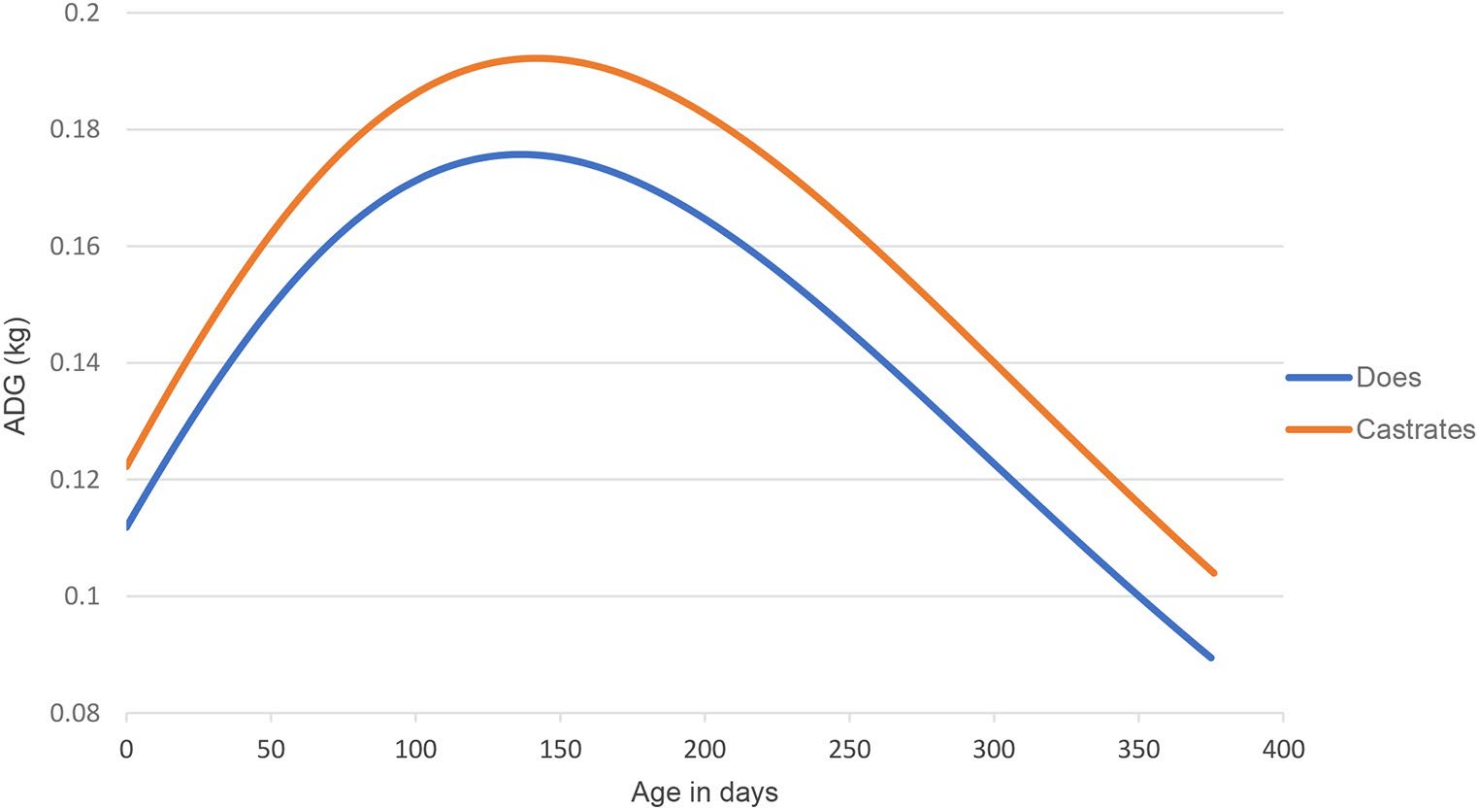
Illustration of the change in the average daily gain (ADG) of Boer goat castrates and does, as predicted by differentiation of the Gompertz growth curve (${W}_{t}=A{e}^{{-e}^{-k(t-c)}}$)

Modelling the growth of goats gives a good indication of a goat’s body weight at a specific age, however, it is also necessary to examine the changes that occur in growth rate over certain time periods. By differentiating the Gompertz growth curve, changes in the growth rate of the goats over time can be determined and visualised (Fig. 1). Growth increases up to the inflection point on the Gompertz growth curve (parameter *C*). When the inflection point is reached, growth decreases as the animals mature. The inflection point can therefore also be seen as the point where maximal growth occurs. Figure 1 depicts the average daily gain of Boer goat castrates and does over the trial period, reaching a maximal growth rate of 0.192 kg/day and 0.175 kg/day, respectively.

**Fig. 2:**
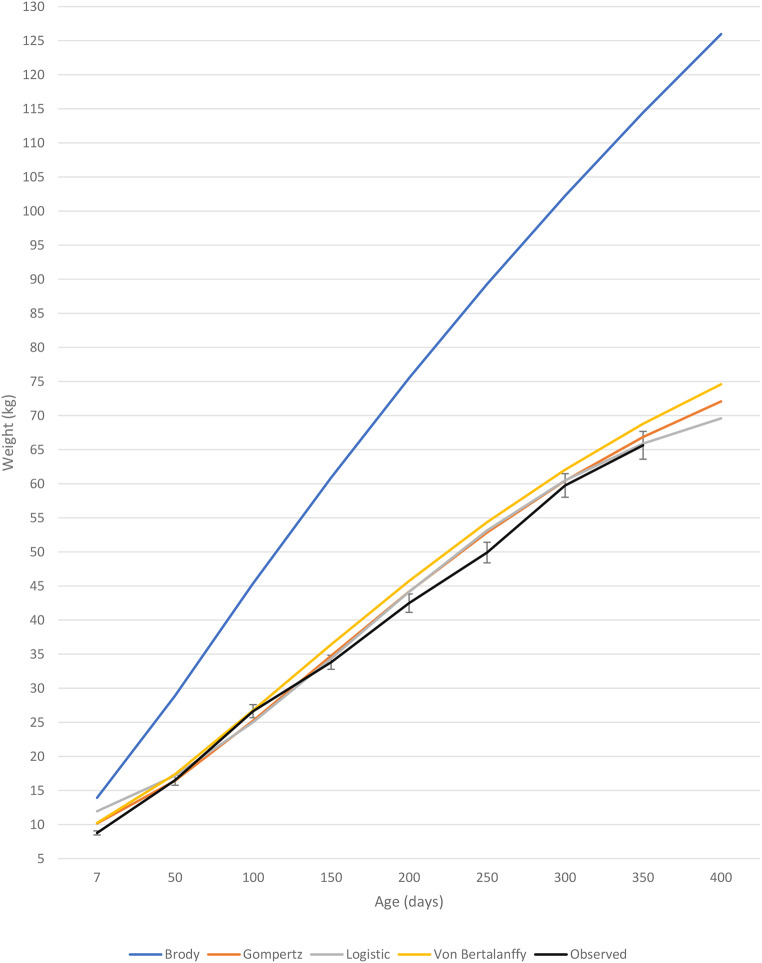
Observed live weights (± SE) of Boer goat castrates up to one year of age, including live weight predictions by the Brody, Gompertz, Logistic and Von Bertalanffy models

**Fig. 3:**
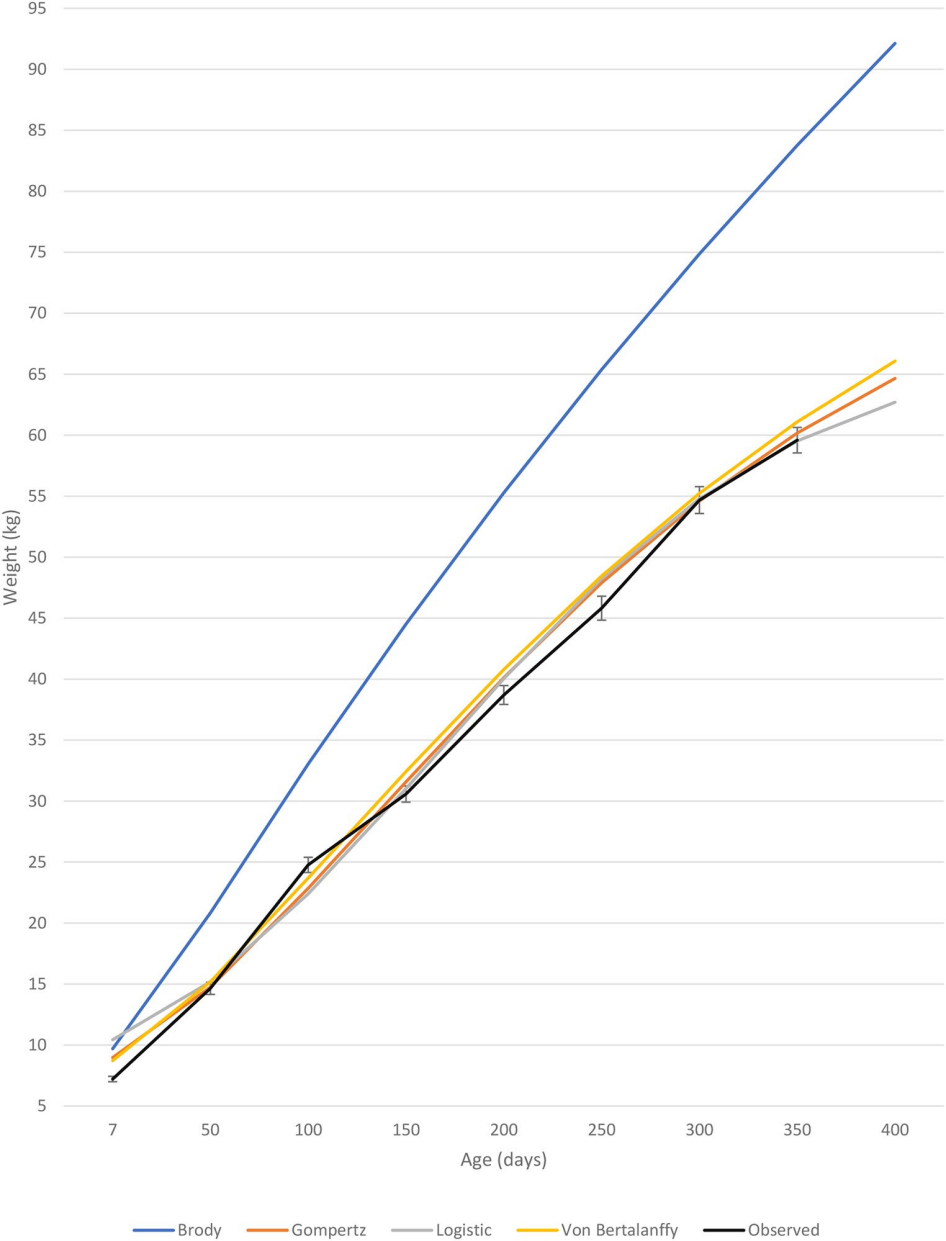
Observed live weights (± SE) of Boer goat does up to one year of age, including live weight predictions by the Brody, Gompertz, Logistic and Von Bertalanffy models

From Figs. 2 and 3, it is evident that the Brody model severely exaggerated the growth estimates for Boer goat castrates as well as does. In contrast, the three remaining growth models closely resembled the mean observed weight of Boer goat castrates (Fig. 2) and does (Fig. 3). While the Von Bertalanffy model seemed to describe the growth of Boer goat castrates well (Table 5), upon inspection of a graphical representation, it is evident that the model overestimated body weight during the entire lifetime of the castrates. All the growth models were found to overestimate birthweight and early growth of Boer goat castrates and does. It is suggested that the Gompertz model be used for the most accurate representation of early growth. The Von Bertalanffy model most accurately predicted weaning weight for Boer goat castrates and does, while the Gompertz and Logistic model slightly underestimated weaning weight for both sexes. The Logistic model predicted the most accurate year-old weights for Boer goat castrates and does, while the Gompertz model slightly overestimated year-old weights for both sexes. Although the growth models show no significant difference between maturation rate between the sexes, Figs. 2 and 3 provides a visual representation of the superior growth rate and higher mature weight experienced by castrates.

## Discussion and conclusion

In the current study, the Gompertz and Von Bertalanffy functions were found to describe Boer goat castrates’ growth the best as lower RMSE and AIC values are reported, with high R^2^ values compared to the other models for castrates (Table 5). The Gompertz model had a slightly higher RMSE and AIC than the Von Bertalanffy model but had a similar R^2^ which advocates the Gompertz model being appropriate for describing Boer goat castrates’ growth. The Gompertz function fits the growth of Boer goat does the best, reporting the lowest RMSE and AIC of all four growth models for does and equal highest R^2^ value (Table 5). The Brody model was not considered suitable for describing the growth of Boer goats as the predicted asymptotic mature weight (*A*) was noticeably higher than all the other models and greatly exaggerated (Table 5). The mature weights for Boer goat bucks and does are reported as 90–130 kg and 80–100 kg, respectively (Lu 2001). Thus, the use of the Brody model could lead to inaccurate weights being predicted.

Parameter *A* provides an indication of the asymptotic mature weight of the Boer goat castrates and does. Larger mature weights imply that the animal is heavier at maturity and thus will have a slower maturation rate. The Gompertz model predicted mature weights of 90.2 kg for Boer goat castrates and 79.3 kg for does (Table 4). This significant difference in mature weight has previously been reported by Steinbach (1987), and the prevalence of sexual dimorphism in the mature weight of Boer goats was also ascribed as being greater in Boer goats than other goat species. Producers often use Boer goat bucks as the sire breed in crossbreeding schemes as they provide a better increase in growth rate for the progeny than when Boer goat does are used, as the maternal effects are described as being mostly negative (Angwenyi and Cartwright 1984). Snyman (2014) described the mature weight of Boer goat does to be 80 kg, which is very similar to the mature estimate predicted by the Gompertz function. It seems that the Logistic function underestimated asymptotic mature weight, while the Von Bertalanffy and Brody functions have overestimated the asymptotic mature weight to be reached by Boer goat castrates and does (Table 4). Garcia-Muniz et al. (2019) found the mature weight for the does of Boer goats from the Mexican breeding flock to be estimated at 54.7 kg from the Gompertz equation and 57.4 kg from the Von Bertalanffy model, which is lower than the estimated asymptotic mature weight for this study. Similarly, Ariff et al. (2010) found that the Gompertz and Von Bertalanffy model estimated the mature weights of Boer goat does from Malaysia to be 58.23 kg and 59.31 kg, respectively. A possible explanation for the higher mature weights observed in the current study, compared to the two previously mentioned studies, is the use of an *ad lib* feedlot diet. The diet used in the current study was supplied to allow animals to achieve their maximum genetic growth potential. Therefore, it can be expected that higher mature weights would by obtained. These differences in mature weights may also be ascribed to the genetic differences between Boer goat populations of different countries. The asymptotic mature weight of Boer goats is also much heavier than other species of goats. The Akkeci goat from Turkey has been shown to have an asymptotic mature weight of 48.4 kg using the Gompertz equation (Kor et al. 2006). Gautam et al. (2019) found the asymptotic mature weight of the dual-purpose Sirohi goat from India to be 26.9 kg for males and 24.1 kg for females using the Gompertz model. In contrast, Sunwasiya et al. (2019) estimated the mature weight for the breed to be 26.2 kg using the Brody function.

The *k*-parameter represents maturation rate. The maturation rate is an indication of the rate at which the mature weight is gained from birth. Animals with higher maturation rates reach maturity earlier because they experience their maximal growth rate at a younger age than those with a lower maturation rate (Owens et al. 1993). Thus, the earlier maturing animals are not likely to experience heavy mature weights (da Silva et al. 2012). Malhado et al. (2009) suggested that higher maturation rates indicate a faster growth rate. In the present study, all the growth models showed a heavier mature weight for castrates than does (Table 4). This relationship is expected among sexes of the same breed as females commonly mature earlier than their male counterparts. Due to sexual dimorphism and heavier mature weights of the goat castrates, it is expected that they will achieve a higher growth rate than the does (Butterfield 1988; Owens et al. 1993). However, no difference (*P* > 0.05) was detected between the maturation rate (parameter *k*) of Boer goat castrates and does in this study and thus, it can be assumed that the maturation rates are relatively similar (Table 4). Normally there is a negative relationship between mature weight (parameter *A*) and maturation rate (parameter *k*) (Malhado et al. 2009; Bathaei and Leroy 1998). This negative correlation indicates that when producers select for higher mature weights, it will result in offspring with slower maturation rates and vice versa.

The inflection point of a growth curve represents the age where an animal experiences the maximal growth rate. After an animal reaches the inflection point, the growth rate declines as the animal reaches maturity and from a producer’s perspective, it is no longer beneficial to feed and grow the animal. Inflection points are found in the Gompertz, and von Bertalanffy models at around 37%, and 30% of the mature weight (parameter *A*), respectively (Teleken et al. 2017). It was postulated by Goshu and Koya (2013) that the Logistic model’s inflection point could be calculated by halving the asymptotic mature weight (parameter *A*). By contrast, the Brody model lacks an inflection point (Teleken et al. 2017). The Gompertz equation, as proposed by Emmans (1989), was used in this study as the *C*-parameter can be biologically interpreted as the inflection point. In this form, the *C*-parameter denotes the age at which maximal growth occurs. Furthermore, the animal’s weight at the inflection point can be determined (Emmans 1989). When the animal’s age is equal to the age predicted by the inflection point (parameter *C*), the animal’s weight at the inflection point can be calculated by simply dividing the mature weight (parameter *A*) by Eulers constant (*A*/*e*).

The Boer goat boasts high growth rates and fecundity compared to other goat breeds and is globally regarded as the best meat-producing goat breed (Steyn 2010). Boer crossbred kids exhibit slower growth than pure Boer goats, but studies have reported growth rates of 150–160 g/day (Cameron et al. 2001). In a meta-analysis on 11 publications done by Luo et al. (2004) on dairy and meat (≥ 50% Boer) goats, the authors found growth rates of 158 g/day for meat-type goats and 138 g/day for dairy-type goats. Male animals are also generally superior to female animals of the same breed in terms of growth rate (Van Niekerk and Casey 1988; El Muola et al. 1999), as is seen in Fig. 3 with a higher growth rate exhibited by castrates than does. In this study Boer goat castrates had an average daily gain above 150 g/day from 13.34 kg up to 57.55 kg body weight, whereas the Boer goat does only have an average daily gain above 150 g/day from 14.75 kg till 46.42 kg body weight. Boer goat castrates exhibited a maximal growth rate of 192 g/day while does have a maximal growth rate 175 g/day. However, it has been shown that under favourable feedlot conditions Boer goats can exhibit ADGs as high as 200 g/day (Sheridan et al. 2003; Brand et al. 2020). Brand et al. (2020) reported average daily gains of 192.2 g/day, 216.8 g/day and 197.2 g/day for Boer goats on low (11.3 MJ ME/kg feed), medium (12 MJ ME/kg feed) and high (12.7 MJ ME/kg feed) energy diets, respectively. The energy content of the diet used in the current study resembles the low energy diet used by Brand et al. (2020) and similar average daily gains are reported. However, in the study by Brand et al. (2020) Boer goats were only reared to 266 days of age with the highest end weights reported for the medium energy diet (49.6 kg). In the present study Boer goat castrates had surpassed this weight at 250 days of age (49.9 kg) and it can thus be assumed that up until 266 days of age the Boer goats in the present study had higher growth rates on a diet with a lower energy content. The growth rates attained in this study are at the top end of those found in literature and thus indicate that the growth rate of Boer goats can be improved by using feedlot finishing with a diet that satisfies the animal’s nutritional requirements. This supports the statement of Owens et al. (1993) that although an animal’s genetics ultimately dictate the mature weight and potential growth rate of the animal, nutrition can be used to advantageously manipulate these factors.

Higher growth rates are experienced at younger ages due to young animals having a higher proportion of muscle while also depositing fewer amounts of fat (Owens et al. 1993). As the animal ages, protein is deposited in muscle and fat in adipose tissue (Lawrence et al. 2012). To allow for muscle and fat tissue growth, enough metabolizable energy needs to be supplied in the diet. Diets typically supply more metabolizable energy than required for the development of just muscle which facilitates the deposition of fat (Webster 1980). Less energy is required to deposit protein for the growth of muscle than the energy required for the deposition of fat since fat is more energy-dense than muscle (Webster 1980). To increase an animal’s average daily gain, the animal’s voluntary feed intake must exceed the animal’s maintenance requirements. According to Owens et al. (1993), an animal’s growth rate plateaus as the animal reaches the mature bodyweight because the amount of energy required for maintenance increases with increased tissue mass. Growth rate reaches a maximum as the animal matures and after this point growth rate declines. The deterioration in growth rate is owing to the increased cost for maintenance in the form of energy, which is associated with older animals, and thus less energy is available for production. After a mature mass has been attained, only dietary energy ingested above the maintenance requirement will increase production through the deposition of fat. Maximum muscular mass is acquired when an animal reaches maturity (Owens et al. 1993). In Fig. 3, this decline in growth rate can be seen as the animals start reaching maturity at 150 days of age. This corresponds to the inflection point predicted by the Gompertz equation for Boer goat castrates and does (Table 4).

As previously mentioned, growth can only occur when energy is consumed above the maintenance requirement of the animal. When animals receive feed in *ad-lib*, intake will increase in proportion to the animal’s size. As animals get older, the gastrointestinal tract matures and physically restricts the amount of feed that can be ingested. Furthermore, the greater increase of fat depositions associated with high energy diets (Ryan et al. 2007) results in free fatty acids being released in the blood as a feedback mechanism that suppresses hunger and leads to a decrease in feed intake (NRC 1987). Consequently, the animal will experience a decrease in growth rate as maturity is reached and the maintenance costs of the animal increases resulting in less available energy for production (Owens et al. 1993; Lawrence et al. 2012).

To produce Boer goats, growth rate and bodyweight are the most economically important features. Thus, a good understanding of these traits is beneficial to producers and should be incorporated in management strategies. Based on goodness of fit statistics, the Von Bertalanffy and Gompertz functions were deemed the most appropriate models to describe Boer goat castrates’ growth, while the Gompertz model described the growth of Boer goat does the best. The Gompertz, Logistic and Von Bertalanffy models also predicted significantly heavier weights for Boer goat castrates than for does. Even though the observed and expected curves matched well, the curve should be interpreted cautiously as the Gompertz model underestimated weaning weight and overestimated yearling weight. The current study boasts a very high record keeping rate, with animal weights being recorded once a week for a year, while similar studies usually have a much lower recording rate. Differentiation of the Gompertz growth curve provided insight into the change of growth rate of growing Boer goat castrates and does, and the inflection point provided an indication of the maximum growth rates that can be attained. These models represent the growth of Boer goat castrates and does under feedlot conditions and thus optimal growth conditions. By appropriately applying the growth models depicted in this study, it is possible to develop a model to simulate the growth of Boer goat castrates and does. The use of this model in a precision livestock rearing system will allow producers to estimate the growth of Boer goat castrates and does at certain ages and weights. These estimates can be utilized to establish a baseline for comparisons between animals and ascertain optimal rearing and weights and ages.

## Data Availability

The data sets generated during the current study are available from the corresponding author upon reasonable request.
